# The ugly face of deep vein thrombosis: Phlegmasia Cerulea Dolens—Case report

**DOI:** 10.1016/j.ijscr.2019.05.021

**Published:** 2019-05-11

**Authors:** Ayman S. ELsaid, Abdullah Saleh AlQattan, Ehab Elashaal, Humood AlSadery, Ibrahim AlGhanmi, Bander Fuhaid Aldhafery

**Affiliations:** a Department of General Surgery King Fahad University Hospital, Imam Abdulrahman Bin Faisal University, Saudi Arabia; b Medical Intern, King Fahad University Hospital, Imam Abdulrahman Bin Faisal University, Saudi Arabia; c Demonstrator, Department of General surgery - Division of Vascular Surgery, King Fahad University Hospital, Imam Abdulrahman Bin Faisal University, Saudi Arabia; d Department of Radiology King Fahad University Hospital, Imam Abdulrahman Bin Faisal University, Saudi Arabia

**Keywords:** Deep vein thrombosis, Endovascular procedures, May turner syndrome, Phlegmasia cerulean dolens

## Abstract

**Introduction:**

Phlegmasia Cerulea Dolens (PCD), a rare & life-threatening condition caused by a massive deep venous thrombosis that is associated with arterial occlusion caused by the subsequent compartment syndrome.

**Case presentation:**

A 56-year-old male was diagnosed as a case of extensive left femoral DVT & pulmonary embolism. Two days after being managed by systemic thrombolytics & heparin, his condition worsened as he developed cyanosis of the affected limb, compartment syndrome & foot drop so he was referred to our facility for further management. CT venogram showed a thrombosis of the left popliteal vein extending into the left common iliac vein confirming the diagnosis of PCD & May-Turner syndrome. We adopted a limb preserving approach using a pharmacomechanical catheter directed thrombolysis (PCDT). The patient recovered fully with a complete resolution of his foot drop.

**Discussion:**

Several treatment options have been suggested to improve the outcomes of PCD, but due to the rarity of this condition a gold standard treatment is still controversial. But regardless of the chosen approach, there is an urgent need to decrease the thrombus burden to prevent further adverse sequelae like amputation or even death which can be achieved by using PCDT as it was demonstrated in our case.

**Conclusion:**

Our case shows that a rare entity of DVT as PCD could be a result of improper management of acute proximal DVT in the background of anatomical variabilities & that despite the late presentation of such a rare condition there still a role for a limb preserving approach with endovascular techniques.

## Highlights

• Phlegmasia Cerulea Dolens, a rare & life-threatening condition caused by a massive DVT that is associated with arterial occlusion caused by the subsequent compartment syndrome.
• Phlegmasia Cerulea Dolens could be a result of improper management of acute proximal DVT in the background of anatomical variabilities like May-Turner syndrome.
• Despite the late presentation of such a rare condition there still a role for a limb preserving approach with endovascular techniques.

## 1 Introduction

The following work has been reported in line with the SCARE criteria [1].

Phlegmasia Dolens (PD) is a rare limb & life-threatening disorder that is caused by a massive venous thrombosis of extremities that is associated with acute limb ischemia in the severe cases. Phlegmasia Dolens is sub classified into: 1-Phlegmasia Alba Dolens (PAD) which is a milder, non-ischemic (patent arterial supply) form of PD where there is a subtotal occlusion of the affected vein and intact collateral venous system 2-Phlegmasia Cerulea Dolens (PCD) which is the worst clinical scenario of venous thrombosis that a patient can present with aside from a massive pulmonary embolism [2,3]. PCD is where there is a total occlusion of the affected vein, impaired collateral venous system with arterial occlusion caused by the subsequent compartment syndrome. Hence, these patients present with a classical clinical triad of; intense pain out of proportion, significant edema & cyanosis of the affected limb. From which comes the name phlegmasia cerulean dolens (blue, painful leg). It is associated with a high risk of amputation (50%), pulmonary embolism (22%) and a mortality rate up to 40% [4,5]. Because of the different involvement of the venous and arterial systems of the affected limb in Phlegmasia Cerulea Dolens & Phlegmasia Alba Dolens the presentation, management & prognosis of each one of them is also different from each other. Several treatment options have been suggested to reduce the high rate of morbidity & mortality, but due to the rarity of this condition a gold standard treatment plan is still controversial [3].

## 2 Case presentation

**Fig. 1:**
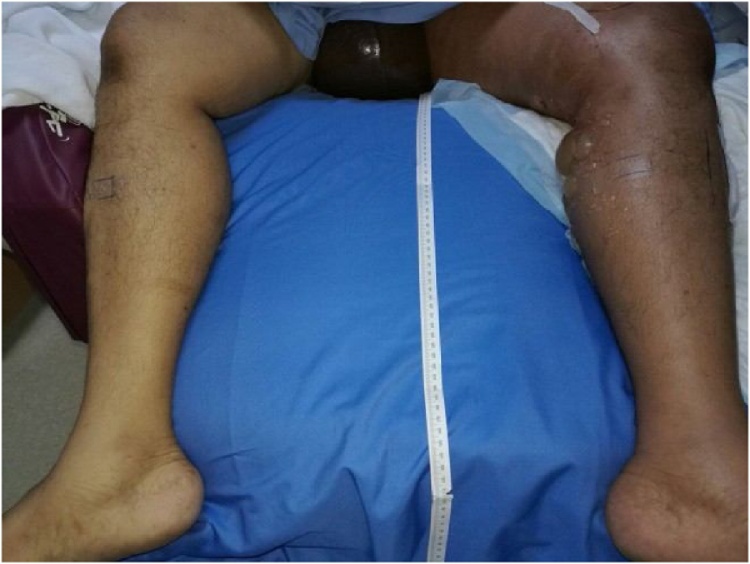
PCD at time of presentation.

We report a case of a 56-year-old male medically free with a history of long travel two days prior to his presentation to another hospital complaining of: left leg swelling, pain & shortness of breath of 2 days duration where he was diagnosed as a case of extensive deep left femoral vein thrombosis & pulmonary embolism. He was kept on systemic thrombolytic therapy & heparin. Two days later, the patient condition started to deteriorate so he was referred to our facility for further management. When he presented to our facility he was in respiratory & pain distress, but hemodynamically stable. Left Lower limb examination showed (Fig. 1): cyanosis, severe edema, blistering of skin extending up to the scrotum. Also there was severe tenderness all over the limb with exacerbation of pain on passive stretching of anterior compartment & left foot drop. CT angiogram of chest & CT venogram lower limbs showed: Extensive thrombosis of the left popliteal vein extending to the left common iliac vein till beginning of the inferior vena cava & pulmonary embolism. The patient was diagnosed as a case of 'Phlegmasia Cerulea Dolens’ of left leg, compartment syndrome with pulmonary embolism (PE).

**Fig. 2:**
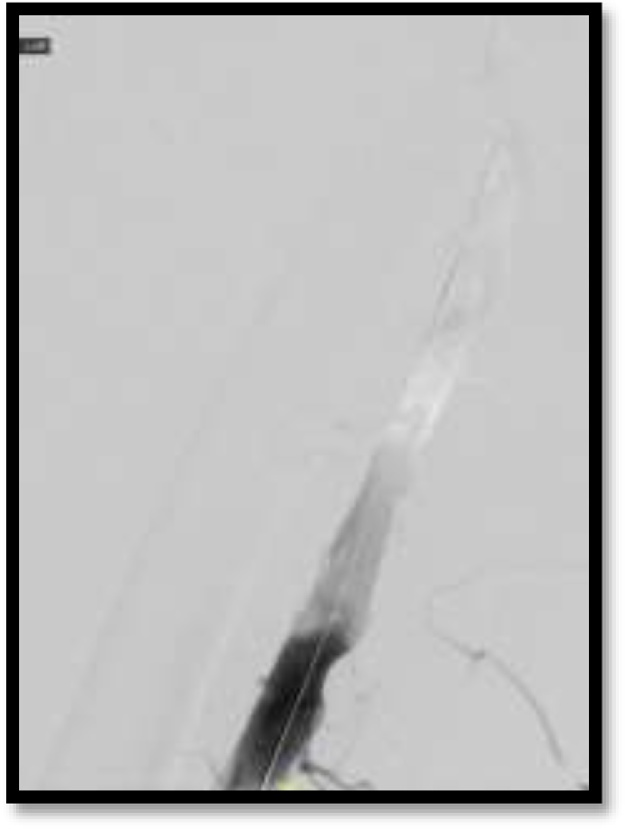
Venogram Pre PCDT.

**Fig. 3:**
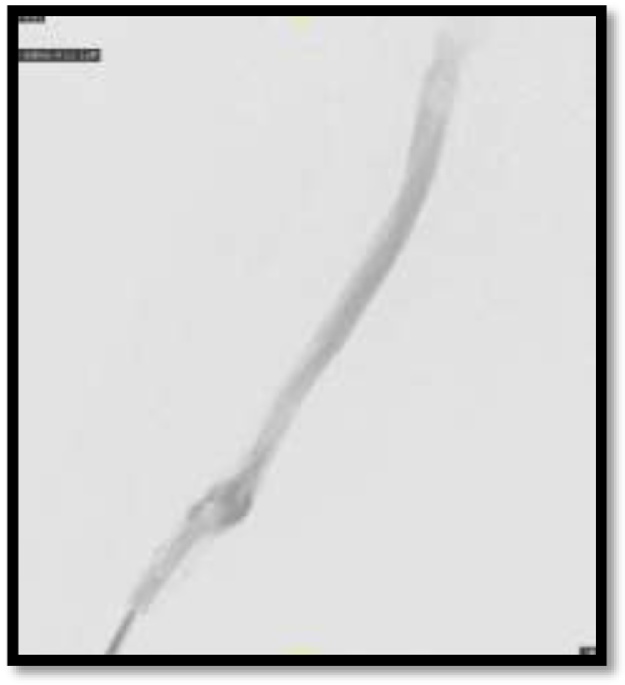
Venogram during PCDT.

**Fig. 4:**
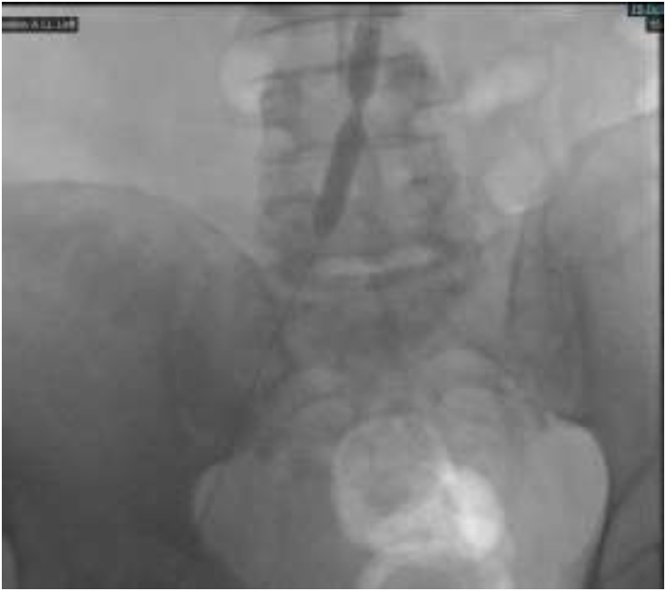
May-Turner syndrome during venogram (in prone position).

**Fig. 5:**
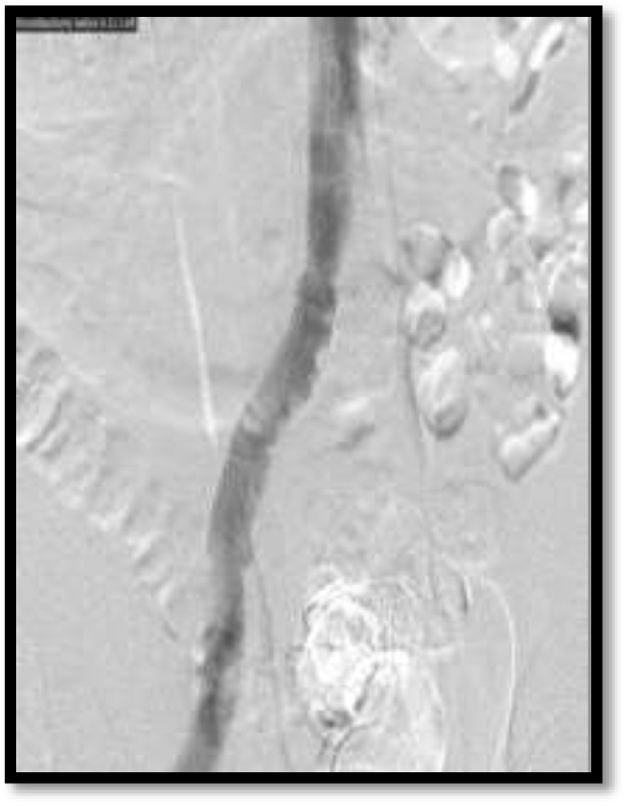
Venogram post PCDT & stenting.

So, fasciotomy was done then he was shifted to the angio suite for pharmaco-mechanical thrombolysis. Under ultrasound guidance & putting the patient in a prone position, the occluding thrombus was accessed distally from popliteal vein & a retrograde venogram was done, which showed a thrombus that almost completely occluding the popliteal vein (Fig. 2) extending all the way up to ilio-femoral veins with no contrast passing through to the inferior vena cava (I.V.C). Subsequently a hydrophilic guide wire was passed through the thrombus into the I.V.C. followed by infusion catheter delivering the tPA & heparin infusion to the sheath was established. Then the patient was shifted to the ICU for monitoring. After 18 h, the patient was brought back to the angio suite & a venogram was done which showed lysis of most of the thrombus burden (Fig. 3). The remaining thrombus was cleared with mechanical thrombectomy catheter. However, there was narrow segment in the left common iliac vein at the confluence (Fig. 4). Intravascular ultrasound (IVUS) revealed a compression of the vein by the crossing of left common iliac artery making the diagnosis of May-Turner syndrome. Then the vein was stented with a size 16mm, length 21mm self-expandable wall stent with no residual stenosis left Fig. 5). Subsequent session of debridement of necrotic tissue of the affected compartment post fasciotomy was done and a negative-pressure wound therapy was adopted to improve the wound healing.

Then the wound was closed by delayed primary closure. The patient condition continued to improve, the arterial system became intact (clinically & radiologically), and he was discharged with anticoagulant (NOAC) and follow-up with physiotherapy to manage his partial foot drop, which was resolved completely later on. A coagulation profile has been done for our patient which turned to be negative. Hence, the cause of our patient condition is most likely 2ry to the prolonged travailing with the coexisting May---Turner syndrome.

## 3 Discussion

Several treatment options have been suggested to improve the outcomes of PCD, but due to the rarity of this condition a gold standard treatment is still controversial. Yet the general initial management should include absolute bed rest, affected leg elevation, fluid resuscitation, and the intravenous heparin. Anticoagulation alone has no role in removing the existing thrombus, as the anticoagulant role is to prevent the progression of the thrombus & the formation of a new one [2,6]. The next step in the management is where controversy lies as there are five approaches of treatment each with its own advantages and disadvantages they are: 1-Surgical thrombectomy, 2-Percutaneous manual aspiration thrombectomy (Mechanical), 3-Systemic thrombolytics (systemic pharmacological), 4-Catheter directed thrombolytics (Localized pharmacological) 5-Pharmaco-mechanical catheter directed thrombolysis (PCDT) [3,7]. Systemic thrombolytics are discouraged to be used as it carries a risk of systemic bleeding & from the surgical site of the fasciotomy. Also, there is a 50% chance of developing Post-thrombotic syndrome resulting in poor quality of life. Manual aspiration thrombectomy for treatment of PCD has been reported by L. oguzkurt et al where it as it was used in a patient with heparin-induced thrombocytopenia and recurrent thrombosis after the initial Manual aspiration making it useful to avoid the use of any thrombolytic in patients with such coagulopathies [4,7] On the other hand, minimally invasive catheter-directed interventions which includes: 1) Catheter-Directed Thrombolysis (CDT) which works by inserting a catheter subcutaneously to reach to the occluding thrombus to deliver high concentration of a thrombolytic agent proximal to the thrombus reducing the risk of systemic bleeding but the downside of using it is the prolonged infusion of the thrombolytic agent which needs to be monitored in the ICU. 2) Pharmaco-Mechanical technique which combined both mechanical fragmentation (to create tracks within the thrombus) and infusion of thrombolytic agent to lyse the thrombus. There are several catheters in the market that are FDA approved for this indication. Most of them share the same basic principles of fragmentation & aspiration of thrombus. The addition of the mechanical effect of the PCDT to the CDT augmenting the effect of the localized thrombolytic thus the faster removal of the thrombus which is critical in the case of PCD to prevent the gangrenes stage of PCD. The American College of Chest Physicians (ACCP) recommended consideration of catheter-directed thrombolysis in selected patients with extensive acute proximal deep venous thrombosis who have low risk of bleeding (Grade 2B level of evidence) [8]. As the utilization of these minimally invasive catheters interventions have several advantages over the systemic ones as they have no systemic effect hence, the less chance of bleeding & risk of developing PE. When it comes to choosing between CDT and PCDT, the later was associated with lower thrombolytic doses, better removal of the thrombus and there for a better flow and less chance for developing venous gangrene (reducing the 50% chance into 25%) & shorter ICU and hospital stays [6]. Although there is an evidence from the randomized controlled 'acute thrombosis: thrombus removal with adjunctive catheter directed thrombolysis (ATTRACT) trial" which revealed that the addition of catheter-based intervention to standard of care anticoagulation in patients with acute proximal deep-vein thrombosis didn't significantly decrease the occurrence of post-thrombotic syndrome [8,9]. But in the management of complicated cases as in PCD there is an urgent need to decrease the thrombus burden to prevent further sequel of the PCD like ischemic necrosis that necessitate the need for amputation or even death in more severe cases or delayed management of such cases which can be avoided by using PCDT as it was demonstrated in our case. The last treatment modality is the open surgical thrombectomy, its main advantage is the instant relief of the occlusion but it is a difficult procedure that requires the use of general anesthesia which might propose a challenge for patients with co-morbidities. The surgical approach was also was found to be associated with higher rate of: recurrence of thrombus,and mortality compared to the interventional catheters. In patients presenting with acute ilio-caval thrombosis or its complicated form (PCD) in the background of May-Thurner Syndrome placing an iliocaval stent should be consider to decrease the recurrence of thrombus thus decreasing the risk of developing post thrombotic syndrome [10,11].

## 4 Conclusion

Phlegmasia Cerulea Dolens is the worst form of deep vein thrombosis. It could develop after an acute proximal DVT & carries a significant risk of mortality & morbidity. An early detection and appropriate decision regarding the line of management is crucial to save the patient and to preserve the limb and its function.

## Conflicts of interest

The authors have no conflicts of interest.

## Sources of funding

None.

## Ethical approval

Case reports are exempted from ethical approval according to policies of Imam Abdulrahman Bin Faisal University.

## Consent

Written informed consent was obtained from the patient for publication of this case report

## Author contribution

Ayman ELsaid: writing the paper, reviewing final manuscript

Abdullah Saleh AlQattan: writing the paper, reviewing final manuscript

Ehab Elashaal : writing the paper, reviewing final manuscript

Humood AlSadery: literature review & writing the paper

Ibrahim AlGhanmi : writing the paper

Bander Fuhaid Aldhafery : writing the paper, reviewing final manuscript

## Registration of research studies

Doesn't apply.

## Guarantor

Ayman ELsaid

Abdullah Saleh AlQattan

## Provenance and peer review

Not commissioned, externally peer-reviewed.
